# Time on wait lists for coronary bypass surgery in British Columbia, Canada, 1991 – 2000

**DOI:** 10.1186/1472-6963-5-22

**Published:** 2005-03-14

**Authors:** Adrian R Levy, Boris G Sobolev, Robert Hayden, Michael Kiely, J Mark FitzGerald, Martin T Schechter

**Affiliations:** 1 Department of Health Care and Epidemiology, University of British Columbia, Vancouver, Canada; 2 Centre for Health Evaluation & Outcome Sciences, St. Paul's Hospital, Vancouver, Canada; 3 Centre for Clinical Epidemiology and Evaluation, Vancouver General Hospital, Vancouver, Canada; 4 Department of Surgery, Royal Columbian Hospital, Vancouver, Canada; 5 British Columbia Cardiac Registries, St. Paul's Hospital, Vancouver, Canada

## Abstract

**Background:**

In British Columbia, Canada, all necessary medical services are funded publicly. Concerned with growing wait lists in the mid-1990s, the provincial government started providing extra funding for coronary artery bypass grafting (CABG) operations annually. Although aimed at improving access, it is not known whether supplementary funding changed the time that patients spent on wait lists for CABG. We sought to determine whether the period of registration on wait lists had an effect on time to isolated CABG and whether the period effect was similar across priority groups.

**Methods:**

Using records from a population-based registry, we studied the wait-list time before and after supplementary funding became available. We compared the number of weeks from registration to surgery for equal proportions of patients in synthetic cohorts defined by five registration periods in the 1990s.

**Results:**

Overall, 9,231 patients spent a total of 137,126 person-weeks on the wait lists. The time to surgery increased by the middle of the decade, and decreased toward the end of the decade. Relative to the 1991–92 registration period, the conditional weekly probabilities of undergoing surgery were 30% lower among patients registered on the wait lists in 1995–96, hazard ratio (HR) = 0.70 (0.65–0.76), and 23% lower in 1997–98 patients, HR = 0.77 (0.71–0.83), while there were no differences with 1999–2000 patients, HR = 0.94 (0.88–1.02), after adjusting for priority group at registration, comorbidity, age and sex. We found that the effect of registration period was different across priority groups.

**Conclusion:**

Our results provide evidence that time to CABG shortened after supplementary funding was provided on an annual basis to tertiary care hospitals within a single publicly funded health system. One plausible explanation is that these hospitals had capacity to increase the number of operations. At the same time, the effect was not uniform across priority groups indicating that changes in clinical practice should be considered when adding extra funding to reduce wait lists.

## Background

Patient access to care within a certain time is an important performance indicator of health systems[1,2]. In publicly funded health care, wait lists are commonly used to manage access to elective procedures raising concerns about delaying necessary treatment[3,4]. In patients with coronary artery disease (CAD) requiring coronary artery bypass graft (CABG) surgery, delaying the operation may lead to deterioration in the patient's condition, worsening of clinical outcomes and increased risk of death[5,6]. Queuing CAD patients according to urgency of treatment is generally perceived as a method of facilitating access to care within clinically appropriate time[6].

In the Canadian province of British Columbia (BC), all medically necessary services are publicly funded[7]. Concerned with growing wait lists for cardiac surgery in the mid-1990s the provincial government started providing supplementary funding to increase the number of CABG operations by 15% annually starting in 1998[8]. Although aimed at improving access, it is not known whether these measures changed the time patients spent on CABG wait lists.

Previous studies showed inconsistent results regarding the impact of supplementary funding on time to surgery in existing hospitals in publicly funded health systems[9]. It has been suggested that the effect may vary according to the scope and the term of funding commitment, such as single hospital versus all the hospitals in a region, and one time versus on-going increases[10]. Although access to surgery from wait lists depends on the assigned priority, there is little information on the impact of supplementary funding across different priority groups.

In this paper we compare the number of weeks between being registered for CABG and undergoing the operation for equal proportions of patients registered in different years before and after the provincial government started providing supplementary funding. In the study period, patients were prioritized according to the established guidelines to expedite access to surgery if they were considered at greater risk of deterioration or death. The specific research questions were: 1) did the period of registration have an effect on the time patients spent on wait lists for CABG? 2) was the period effect similar across priority groups?

We use all relevant records from the provincial population-based registry of CAD patients identified as needing bypass surgery. Primary comparisons are done across synthetic cohorts of patients defined by two-year periods of registration on the wait lists: 1991–92, 1993–94, 1995–96, 1997–98, or 1999–2000.

## Methods

### Data sources

The provincial Cardiac Surgery Registry, a part of BC Cardiac Registries, was created in 1990 to collect data for reporting, planning and research purposes of participating surgeons and hospitals, and the provincial Ministry of Health[11]. The Registry prospectively captures the occurrence and timing of registration, surgery, or removal from the wait lists without surgery, for all patients accepted for cardiac surgery procedures in the four hospitals delivering all adult open-heart surgery services to four million residents of BC.

Between 1991 and 2000, from 15 to 20 cardiac surgeons were performing bypass surgery in BC, with less than 30% turnover. Although cardiac surgeons manage their wait lists independently, they all routinely provide information to the Registry entry modules: surgery registration, operative report, wait-list reconciliation, and discharge summary. When accepting patients on their wait lists, the surgeons document the indication for the procedure as well as the priority for treatment using common criteria (see below).

Once the operation is completed, the operative report containing the procedure and clinical data is entered in the Registry. Patients are removed from the wait lists after undergoing the operation or for other reasons: if they died, declined the operation, accepted surgery from another surgeon, moved away, or switched to medical management. The crude agreement between the Registry and hospital charts for ten demographic and clinical data elements has been estimated at 86%[11].

We deterministically linked the Registry records to administrative databases storing records of all hospital episodes in BC[12]. These records include the dates of admission, procedure and discharge, as well as diagnoses at discharge[13]. These data were used to corroborate the service dates and to identify coexisting medical conditions[14].

### Patients

If angioplasty is not indicated when the cardiologist evaluates the arterial lesions on the coronary angiogram, then a cardiac surgeon is consulted to assess the patients' suitability for CABG. Patients are transferred to an in-patient ward directly from the catheterization laboratory if expedited assessment is necessary. If deemed suitable, these patients wait for CABG in hospital without registration on a wait list. Alternatively, a consultation with the surgeon can be scheduled at a later date. Surgeons register on their wait lists patients who need CABG and for whom the operation can be safely delayed. As in-patients were not added to wait lists, they were not included in analyses of wait-list times.

There were 9,366 records of registration for isolated CABG added to the Registry between January 1991 and December 2000. We excluded 135 records of patients who were: emergency cases (30), removed on the registration date (101), and had missing operating room reports (4). All remaining 9,231 records had either the surgery date or the date and reason of removal from the list without surgery. We restricted the analyses to the first 52 weeks after registration so that 475 (5%) patients remaining on the lists at 12 months were censored. Of those, 167 eventually underwent surgery; seven died; 78 received medical treatment; 104 declined surgery; 17 were transferred to another surgeon or hospital; and 102 were removed for other reasons.

### Priority groups

When assigning priority, all cardiac surgeons in BC apply common guidelines developed in 1990 [see Additional file 1]. Using the location and degree of affected coronary anatomy and symptoms, the guidelines help to: identify patients for whom CABG can increase survival or improve quality of life[15]; classify patients according to urgency of treatment; and assign a maximum recommended waiting time (MRWT). Patients are assigned priority 1 if they require CABG urgently (eg, left main coronary artery stenosis greater than 70%, MRWT three days); priority 2 if there is moderate urgency (eg persistent unstable angina, MRWT six weeks); or priority 3 if there is less urgency (eg intractable chronic angina, MRWT 12 weeks). These guidelines did not undergo any major revisions through the entire period under study.

### Comorbidity

Using the administrative data, coexisting medical conditions were identified using all primary and secondary discharge diagnoses recorded in all hospital discharge abstracts within one year prior to registration[13]. This time frame was chosen in order to capture the presence of chronic diseases that could have affected the waiting time[14].

For each patient, we identified the presence of major and minor comorbid medical conditions present at registration.

### Statistical methods

Waiting times were analyzed as prospective observations beginning at the time of registration. Each subject had a wait-list time calculated in calendar weeks from registration to surgery or removal for other reasons. The cumulative probability of undergoing surgery as a function of wait-list time was estimated using the Kaplan-Meier method[16]. Patients removed from the list for reasons other than surgery were treated as censored observations.

Primary comparisons were done across synthetic cohorts of patients defined by two-year periods of registration on the wait lists. Differences in the distributions of wait-list times across cohorts were examined using the log rank-test[17]. The average weekly surgery rate was calculated by dividing the number of operations by the total number of patient-weeks on the list. The effect size for each registration period was estimated by hazard ratios for surgery derived from a Cox proportional hazards model[18]. Hazard ratios (HR) associated with registration periods evaluated the conditional weekly probability of undergoing CABG relative to the 1991–92 period. The priority groups and the presence of comorbidity at registration were included as independent variables in the Cox model to estimate adjusted effects. Age and sex were entered into the regression models as strata variables to avoid the proportionality assumption on these factors while using the proportional hazards model.

The Clinical Research Ethics Board of the University of British Columbia approved the study protocol.

## Results

In BC in the 1990s, 9,231 patients were registered on wait lists for CABG and spent a total of 137,126 person-weeks waiting. Over the same period, 9,433 patients underwent isolated CABG without registration on wait lists. The most prevalent groups at registration were men (82%), those without major comorbidities (52%), those registered in priority group 2 (70%), patients aged 60–69 (38%) and 70–79 (30%) years, and those registered in 1995–96 (22%), Table 1. The proportion of patients registered in priority group 1 was lowest in 1999–2000 and highest in the 1995–96 cohort, Table 2. The opposite pattern was observed in priority group 3. Of 8,756 patients who left the lists within 52 weeks: 7,991 underwent surgery; 90 died while waiting; 176 received medical treatments; 188 declined surgery; and 311 were removed due to other reasons.

**Table 1:** Characteristics of 9,231 subjects registered for isolated coronary artery bypass surgery in British Columbia, 1991–2000.

**Characteristic**	**N**	**(%)**
Age group (y)		
<50	732	(7.9)
50–59	2005	(21.7)
60–69	3530	(38.2)
70–79	2770	(30.0)
≥ 80	194	(2.1)
Sex		
Women	1634	(17.7)
Men	7597	(82.3)
Urgency at registration		
Priority 1	659	(7.1)
Priority 2	6496	(70.4)
Priority 3	1963	(21.3)
Unknown	113	(1.2)
Major comorbidity at registration		
None	4769	(51.7)
Minor comorbidity	2450	(26.5)
CHF, diabetes, COPD, rheumatoid arthritis, cancer	2012	(21.8)
Registration period		
1991–1992	1724	(18.7)
1993–1994	1889	(20.5)
1995–1996	2010	(21.8)
1997–1998	1888	(20.5)
1999–2000	1720	(18.6)

Abbreviations: CHF – congestive heart failure COPD – chronic obstructive pulmonary disease

**Table 2:** Distribution of subjects registered for isolated coronary artery bypass surgery in British Columbia, 1991–2000, by priority group and registration period

**Registration**	**Priority 1**		**Priority 2**		**Priority 3**	
**period**	**N**	**(%)**	**N**	**(%)**	**N**	**(%)**

1991–1992	116	(6.7)	1221	(70.8)	334	(19.4)
1993–1994	110	(5.8)	1381	(73.1)	388	(20.5)
1995–1996	249	(12.4)	1363	(67.8)	374	(18.6)
1997–1998	117	(6.2)	1327	(70.3)	428	(22.7)
1999–2000	67	(3.9)	1204	(70.0)	439	(25.5)

Note: Excludes 113 subjects with unknown priority

In all registration cohorts combined, the average weekly number of operations was 5.8 (95% confidence interval 5.7–6.0) per 100 patients listed, the median time on the list was 11 weeks (25th percentile 5 weeks; 75th percentile 22 weeks), and the probability of undergoing surgery after 26 weeks on the list, twice the MRWT for priority group 3, was 20%.

As expected, there were significant differences among priority groups, with larger proportions undergoing CABG at every week among more urgent patients (log rank test = 1611.9, P < 0.0001). The average weekly number of operations per 100 patients on the list differed from 20.6 (19.0–22.2) in group 1 to 6.7 (6.5–6.8) in group 2 to 3.3 (3.1–3.4) in group 3. However, considerable variation in wait-list times was observed within each priority group. For instance, although half of group 1 underwent surgery within two weeks and 90% underwent surgery by 12 weeks, the remaining 10% waited another 1 to 32 weeks (total 13 to 44 weeks).

**Figure 1:**
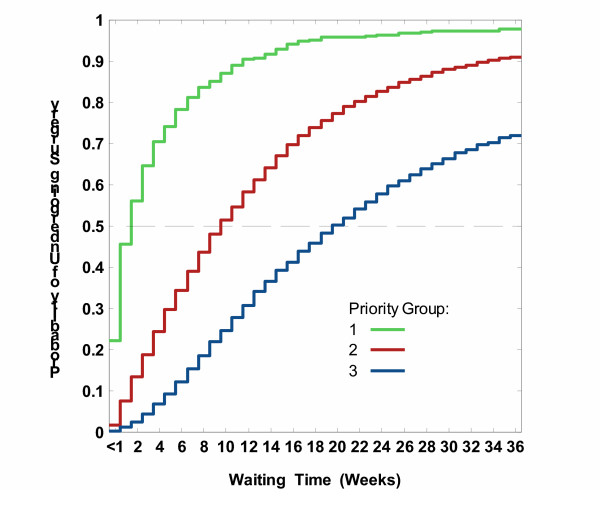
Estimated probabilities of undergoing isolated CABG within a certain time after registration on wait lists, by priority group.

This can be seen in Figure 1, which shows access probabilities for CABG in each priority group. The abscissa shows the number of weeks on the waiting list and the ordinate shows the probability of undergoing operation by that week. Higher probabilities correspond to shorter wait list times. While all patients were removed at 52 weeks, for graphical simplicity we show the first 36 weeks. Access probabilities in priority 3 (blue) were systematically lower indicating longer wait list times than among priority 2 (red line) or priority 1 (green line).

### Access to surgery by registration period

The differences in the proportion of patients undergoing CABG were significant across registration periods (log rank test = 97.3, P < 0.0001), with longer wait-list times for those registered between 1995 and 1998, Figure 2.

**Figure 2:**
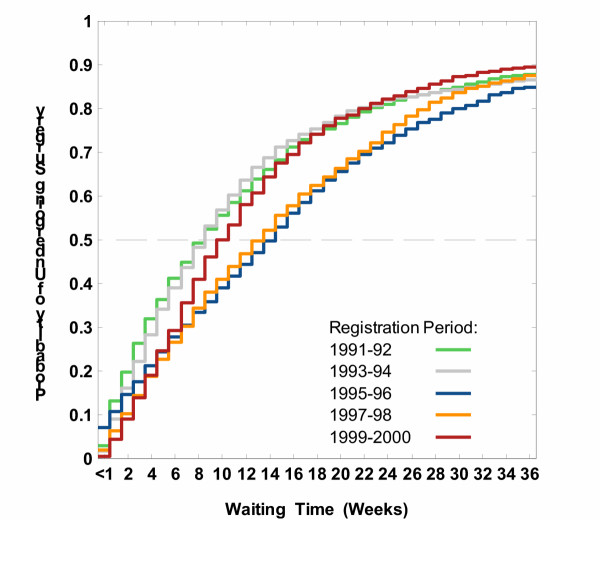
Estimated probabilities of undergoing isolated CABG within a certain time after registration on wait lists, by registration period.

Table 3 shows the number of weeks required for a specified proportion of patients to undergo the operation across registration periods. Wait-list times in 1995–96 were such that 10%, 25%, 50%, and 75% patients underwent surgery within 1, 6, 15, and 26 weeks, respectively, whereas half of the 1991–92 cohort underwent surgery within 9 weeks, and 75% did so within 19 weeks. Comparing the 1995–96, 1997–98 and 1999–2000 cohorts we observed a compression in access to surgery, i.e., reduction in the length of wait-list interval required for a specified proportion to undergo the operation. As measured by the difference between 90^th ^and 50^th ^percentiles of the wait time distributions, 40% of the 1995–96 cohort underwent surgery within 33 weeks following the median time, while it took 29 weeks for the 1999–2000 cohort.

**Table 3:** Percentiles of wait-list time (weeks) for subjects registered for isolated coronary artery bypass surgery in British Columbia 1991–2000 by registration period

**Registration period**	**Percentile**				
	
	**10th**	**25th**	**50th**	**75th**	**90th**
1991–1992	1	3	9	19	44
1993–1994	2	4	9	18	45
1995–1996	1	6	15	26	48
1997–1998	2	6	14	25	43
1999–2000	3	6	10	19	39
All periods	2	5	11	22	44

Note: probability of undergoing outpatient surgery within 26 weeks of registration is 0.804

While the median wait-list time was 11 weeks (the MRWT of priority group 3) in all cohorts combined, 15% of the 1991–92 and 1993–94 cohorts, 22% of the 1995–96 cohort, 19% of the 1997–98 cohort, and 14% of the 1999–2000 cohort experienced an excessive wait, defined as longer than 26 weeks (data not shown). The average weekly number of operations per 100 patients listed varied from 6.5 (6.2–6.9) in the 1991–92 cohort to 5.1 (4.8–5.3) in the 1995–96 cohort to 6.2 (5.9–6.6) in the 1999–2000 cohort, Table 4 (fourth column). Corresponding hazard ratios and 95% confidence intervals (CI) are shown in Table 4, columns 6 and 7. Relative to the 1991–92 cohort, the conditional weekly probabilities of undergoing surgery were 30% lower among 1995–96 patients, HR = 0.70 (0.65–0.76), and 23% lower in 1997–98 patients, HR = 0.77 (0.71–0.83), after adjusting for priority, comorbidity, age and sex. There were no differences between periods 1991–92 and 1999–2000, HR = 0.94 (0.88–1.02).

**Table 4:** Average weekly rate of undergoing the operation from wait list for isolated coronary artery bypass surgery in British Columbia 1991–2000 and adjusted rate ratios by registration period

**Registration period**	**Number of operations**	**Total wait time, weeks**	**Crude Rate, per 100**	**SE**	**Hazard ratio**	95% CI*
1991–1992	1504	23047	6.5	0.2	1.00	referent
1993–1994	1646	25480	6.5	0.2	1.00	0.93, 1.08
1995–1996	1727	34186	5.1	0.1	0.70	0.65, 0.76
1997–1998	1613	30384	5.3	0.1	0.77	0.71, 0.83
1999–2000	1501	24029	6.2	0.2	0.94	0.88, 1.02
All periods	7991	137126	5.8	0.1	-	-

Abbreviations: SE = standard error; CI = confidence interval

*adjusted for priority group and comorbidity; stratified by age and sex

**0 patients were on the wait list on December 31 2001

### Access to surgery by registration period within priority groups

In each priority group, the proportions of patients undergoing CABG at each week on the wait-list was lower among those registered in 1995–96 compared to 1991–92 as measured by log-rank tests (priority 1: chi-square = 5.6, P = 0.0183, 1 df; priority 2: chi-square = 58.2, P < 0.0001, 1 df; priority 3: chi-square = 20.5, P < 0.0001, 1 df). By 1999–2000, the pattern of change was different between priority groups.

In priority group 1, the average weekly number of operations per 100 patients listed declined from 42.4 (34.6–50.2) in the 1991–92 cohort to 20.3 (17.7–22.9) in the 1995–96 cohort to 12.2 (9.2–15.3) in the 1999–2000 cohort (data not shown). Corresponding HRs and 95% CIs are shown in Table 5, columns 2 and 3. The conditional weekly probabilities of undergoing surgery were 34% lower for the 1995–96 cohort, HR = 0.66 (0.50–0.87), and 53% lower for the 1999–2000 cohort, HR = 0.47 (0.33–0.68), relative to 1991–92. There was a difference in the distribution of wait-list times between 1995–96 and 1999–2000 cohorts (chi-square = 9.9, P = 0.0017, 1 df).

**Table 5:** Access to surgery by registration period and priority group for subjects registered for isolated coronary artery bypass surgery in British Columbia 1991–2000, as measured by adjusted hazard ratios*

**Registration period**	**Priority 1**		**Priority 2**		**Priority 3**	
	
	**HR**	**(95% CI)**	**HR**	**(95% CI)**	**HR**	**(95% CI)**
1991–1992	1.00	referent	1.00	referent	1.00	referent
1993–1994	0.79	(0.57, 1.09)	1.10	(1.01, 1.20)	0.78	(0.65, 0.92)
1995–1996	0.66	(0.50, 0.87)	0.71	(0.65, 0.78)	0.69	(0.58, 0.82)
1997–1998	0.49	(0.36, 0.67)	0.82	(0.75, 0.89)	0.75	(0.63, 0.88)
1999–2000	0.47	(0.33, 0.68)	0.99	(0.90, 1.08)	1.07	(0.90, 1.26)

Abbreviations: HR = hazard ratio; CI = confidence interval

*adjusted for priority group and comorbidity; stratified by age and sex

In priority group 2, the average weekly number of operations per 100 patients listed varied from 7.3 (6.8–7.7) in the 1991–92 cohort to 5.3 (5.0–5.6) in the 1995–96 cohort to 7.3 (6.9–7.7) in the 1999–2000 cohort. The adjusted HR in 1999–2000 was 0.99 (0.90–1.08) relative to 1991–92 (Table 5, columns 4 and 5). There was no difference in the distribution of wait-list times between 1991–92 and 1999–2000 cohorts (chi-square = 0.5, P = 0.5, 1 df).

In priority group 3, the average weekly number of operations per 100 patients listed changed from 3.9 (3.4–4.3) in 1991–92 to 2.8 (2.4–3.1) in 1995–96 to 4.0 (3.6–4.5) in 1999–2000. The adjusted HR associated with the 1999–2000 registration period was 1.07 (0.90–1.26) relative to 1991–92 (Table 5, columns 6 and 7). There was no difference between the between 1991–92 and 1999–2000 cohorts (chi-square= 0.6, P = 0.4, 1 df).

## Discussion

In this paper we studied the amount of time that patients with CAD spent on CABG wait lists before and after the provincial government started providing supplementary funding to increase the annual number of CABG operations. We sought to determine whether the period of registration had an effect on the wait-list time and whether the period effect was similar across priority groups. Using the population-based registry, we compared the number of weeks from registration to surgery for equal proportions of patients across different registration periods. In these comparisons, we accounted for the priority mix at registration. We used prospective follow-up of all patients registered to avoid biases inherent in wait-list statistics based on patients undergoing the procedure only[19].

We found that the registration period had an effect on the amount of time that patients spent awaiting CABG in BC in the 1990s. Wait-list times in the 1995–96 cohort were such that 50% and 75% patients underwent surgery within 15 and 26 weeks, respectively, whereas one-half of the 1991–92 cohort underwent surgery within nine weeks and three quarters did so within 19 weeks. This trend was reversed later, such that the 1999–2000 patients waited no longer than did their 1991–92 counterparts. Relative to the 1991–92 cohort, the conditional weekly probabilities of undergoing surgery were 30% lower in 1995–96 patients, and 23% lower in 1997–98 patients, while there were no differences between periods 1991–92 and 1999–2000.

We also found that the effect of registration period was different across priority groups. In priority group 1, the wait-list time increased by the middle of the decade and increased even further by the end of the decade. This may reflect changes in queuing patients with more severe CAD including lessened concern about safety of delaying patients with left main stenosis[20] as well as increasing use of angioplasty to treat patients who would have formerly been treated surgically[21]. In priority groups 2 and 3, the wait-list time also increased by the middle of the decade. In contrast to priority group 1, however, the wait-list time in groups 2 and 3 decreased later, such that there were no differences between the 1999–2000 and 1991–92 cohorts.

Studies examining access to elective care in Canada and elsewhere often report median or mean times [22-25]. We found that reporting the probability of undergoing CABG as a function of wait-list time helps overcome some limitations of using single-value statistics in understanding differences between periods [26-29]. For instance, we were able to conclude that not only did changes in waits reduce the median delay from 15 to 10 weeks in the 1995–96 and 1999–2000 cohorts, respectively, but also provided 20% compression in access for 40% patients staying on the lists longer than the median time. Studying the distributions of wait-list times, we also were able to compare the conditional weekly probability of undergoing CABG across registration periods while adjusting for priority, comorbidity, age and sex.

The lack of information on hospitals or surgeons could be a limitation of this study as we were not able to adjust for the volume of CABG between the four tertiary care hospitals where the operation was performed or for the wait lists between cardiac surgeons.

## Conclusion

Our results provide evidence for a significant reduction in wait-list time after supplementary funding was provided on an annual basis to tertiary care hospitals within a single publicly funded health system. While system-level factors such as changes in the organization or delivery of services may have affected the wait-list time, one plausible reason for the observed reduction was that the hospitals had capacity to increase the number of operations. Compared to 1995–96, there was a 12% increase (from 3,696 to 4,174) in the total number of CABG operations in 1999–2000, Table 6. Also, between 1995–96 and 1999–2000, there was a 13% decrease (from 54% to 41%) in the proportion of patients accessing the operation through wait lists, indicating that supplementary funding was used to provide more operations without delay.

**Table 6:** Distributions of patients who were registered on wait lists or operated without delay in British Columbia 1991–2000, by registration period

**Registration Period**	**Patients identified as needing CABG**			
	
	**Registered on wait lists**		**Operated without delay**	
	
	**N**	**(%)**	**N**	**(%)**
1991–1992	1724	(49.3)	1770	(50.7)
1993–1994	1889	(55.3)	1526	(44.7)
1995–1996	2010	(54.4)	1686	(45.6)
1997–1998	1888	(48.6)	1997	(51.4)
1999–2000	1720	(41.2)	2454	(58.8)

The relatively short time frame following the funding increase is a limitation of our study. As discussed elsewhere, supplementary funding may not result in shortening wait lists if hospitals function near full capacity [30], or, if it is expected that funding will be withdrawn after wait lists are reduced[10]. Reducing wait lists may require investing in new health services facilities. In Denmark, rates of open-heart surgery increased by 70% and the median waiting times declined by half since 1994 when additional capacity for cardiac surgical care was established by increasing the number of operating theatres, equipment and personnel [30]. On-going study of wait-list times for CABG in BC will help determine the permanence of the impact of supplementary funding.

## Competing interests

The author(s) declare that they have no competing interests.

## Authors' contributions

ARL conceived and designed the study, acquired the data, interpreted the results, and drafted the manuscript. BGS conceived and designed the study, analysed the data, interpreted the results, and drafted the manuscript. RH participated in the design of the study, helped acquire the data, and interpreted the results. MK helped acquire the data, and interpreted the results. JMF participated in the design of the study and interpreted the results. MTS participated in the design of the study and interpreted the results. All authors read and approved the final manuscript.

## Pre-publication history

The pre-publication history for this paper can be accessed here:



## Supplementary Material

Additional File 1 The Microsoft^® ^Word 2002 file "BC consensus guidelines for CABG priority.doc" shows the guidelines used by British Columbian cardiac surgeons for assigning priority to patients registered for coronary artery bypass grafting. Click here for file
