# Supplementary material for: Time on wait lists for coronary bypass surgery in British Columbia, Canada, 1991 – 2000
Source: BMC Health Serv Res. 2005 Mar 14;5:22. doi: 10.1186/1472-6963-5-22 (PMC1079832; doi:10.1186/1472-6963-5-22)
Supplement: Additional File 1 — The Microsoft® Word 2002 file "BC consensus guidelines for CABG priority.doc" shows the guidelines used by British Columbian cardiac surgeons for assigning priority to patients registered for coronary artery bypass grafting. [file 1472-6963-5-22-S1.doc]

1. Priority I

The absolute mortality risk per week as observed in the previous 6 years – 0.17%

The median weeks waiting by all patients over the previous 6 months (January 01, 1999-June 30, 1999) – 2.5 weeks

- 1. Left main stenosis > 70%
  2. Hemodynamic instability plus post MI complication
  3. Unstable angina on IV nitroglycerine drip/heparin drip
  4. Syncope
  5. Valve (tissue or native) dysfunction with deteriorating hemodynamics and perfusion
  6. Endocarditis (embolus, vegetation, heart block)
  7. Other symptomatic arrhythmias
  8. Other ­­­­­­­­­­­­­­­­­­­­­­­­­_______________________________________________________

1. Priority II

The abslulte mortality risk per week as observed in the previous 6 years – 0.09%

The median weeks waiting by all patients over the previous 6 months (January 01, 1999-June 30, 1999) – 6.5 weeks

- 1. Persistent unstable angina
  2. Recurrent angina within 6 months of MI
  3. Symptomatic or asymptomatic CAD with
     1. Ominous anatomy-
        1. Left main > 50%
        2. 3-vessel disease with reduced LV Fn or proximal LAD
        3. Left main equivalent
     2. Noninvasive confirmation of ischemia including in patients with double or single vessel disease
     3. Prior ischemia induced VT/VF
  4. Unable to work including as homemaker
  5. Unable to care for dependents
  6. Cardiac tumors
  7. Aortic stenosis with syncope, angina or heart failure
  8. LV aneurysm with failure, embolization, dysrhythmias
  9. Endocarditis with hemodynamic deterioration
  10. Congenital or valvular heart disease with embolization or other symptoms
  11. Cardiovascular foreign bodies
  12. Congenital or valvular lesion with pulmonary hypertention
  13. Asymptomoatic aortic aneurysm
  14. Other _________________________

1. Priority III

The absolute mortality risk per week as observed in the previous 6 years – 0.05%

The median weeks waiting by all patients over the pervious 6 months (January 01, 1999-June 30, 1999) – 8.4 weeks.

- 1. Intractable chronic angina
  2. Subaortic stenosis with evidence of secondary aortic valve deterioration
  3. Critical valvular disease with stable symptoms
  4. Valvular disease with deteriorating or reduced ventricular function
  5. Congenital lesions with stable symptoms
  6. Delayed
     1. Social reasons for delay
     2. Medical reasons for delay
     3. Other reasons for delay ____________
  7. Other ______________________________

Comments:
